# Circular Mini-Incision in the Left Iliac Fossa Followed by Purse-String Closure as a Minimally Invasive Approach for the Sigmoid Volvulus: A Technical Note

**DOI:** 10.7759/cureus.26124

**Published:** 2022-06-20

**Authors:** Ahmed Bensaad, Youssef Ghaddou, Abdelah Nouri, Abdelaziz Fadil, Khalid Sair

**Affiliations:** 1 General Surgery, Cheikh Khalifa International University Hospital, Casablanca, MAR; 2 Surgery, Cheikh Khalifa International University Hospital, Casablanca, MAR; 3 Visceral Surgery, Cheikh Khalifa International University Hospital, Casablanca, MAR

**Keywords:** digestive surgery, gastrointestinal obstruction, mini-open approach, emergency gastroenterology and endoscopy, sigmoid volvulus

## Abstract

Introduction: Volvulus of the sigmoid is a common cause of intestinal obstruction in Morocco. It is a serious condition with substantial mortality. Initial endoscopic decompression followed by resection of the redundant colon via laparotomy or laparoscopy is the procedure of choice. Exteriorization of the sigmoid colon through a linear skin incision in the left iliac fossa has been described as an alternative approach for the classic midline incision, with or without laparoscopic assistance, with acceptable results.

Methods: We describe herein a novel, minimally invasive approach for fit patients with non-complicated volvulus sigmoid. This approach consists of a skin-disk incision in the left iliac fossa, exteriorization of the redundant colon, and resection with or without primary anastomosis, followed by a purse-string closure.

Results: A 65-year-old patient with no prior notable medical history, presented to the emergency department with his first episode of sigmoid volvulus. A skin-disk incision was made in the left iliac fossa, exteriorization of the sigmoid was done easily through the incision, and resection and manual anastomosis were done. The closure was made in a purse-string fashion. Recovery was uneventful and the patient was discharged home on the fourth postoperative day. No wound infection was noted and the cosmetic result was satisfactory.

Conclusion: Left iliac skin-disk incision followed by a purse-string closure is an option for approaching the abdomen in the case of sigmoid volvulus. It has been demonstrated that this technique helps reduce wound-related complications in patients undergoing stoma reversal. Authors suggest that cosmetic results are better and the incidence of surgical site infection can be lower with this technique as compared to the classic linear skin incision. Results, however, should be confirmed by larger studies.

## Introduction

Volvulus of the sigmoid often presents as an acute intestinal obstruction. It is a common finding in developing countries, such as Morocco which has a high reported incidence [1]. The pathophysiology of the intestinal torsion is generally explained by the twisting of redundant sigmoid around its narrow mesentery. Predisposing factors include advanced age, a high-fiber diet, and chronic mental illness [2]. Management of this condition includes initial endoscopic decompression, followed by either midline laparotomy or laparoscopic sigmoidectomy and primary anastomosis.

Approaching the abdomen via left iliac fossa mini-incision has been described in some case reports, with or without prior colonoscopic detorsion [3-5]. As we know from the available data on stoma reversal experience, the purse-string closure technique allows for better cosmetic results and more patient satisfaction than a linear skin incision, and most importantly, it is associated with less surgical site infection incidence [6].

We describe an alternative technique, consisting of a circular skin-disk incision in the left iliac fossa, exteriorization of the redundant colon, and resection with or without primary anastomosis, followed by a purse-string closure.

## Technical report

In this technical note, we describe a 65-year-old patient with no prior notable medical history, who presented to the emergency department with his first episode of sigmoid volvulus. The clinical scenario was consistent with acute colonic obstruction from sigmoid volvulus, with no clinical or radiological signs of complications.

The patient was placed in the supine position and general anesthesia was induced. We made an excision of a circular skin disk in the left-iliac fossa of approximately 3cm in diameter.

With the help of small retractors, we separated the subcutaneous tissue until the exposition of the external oblique aponeurosis. An incision was then made in the direction of its fibers. Muscles were then separated. The careful opening of the peritoneum was made with scissors.

**Figure 1:**
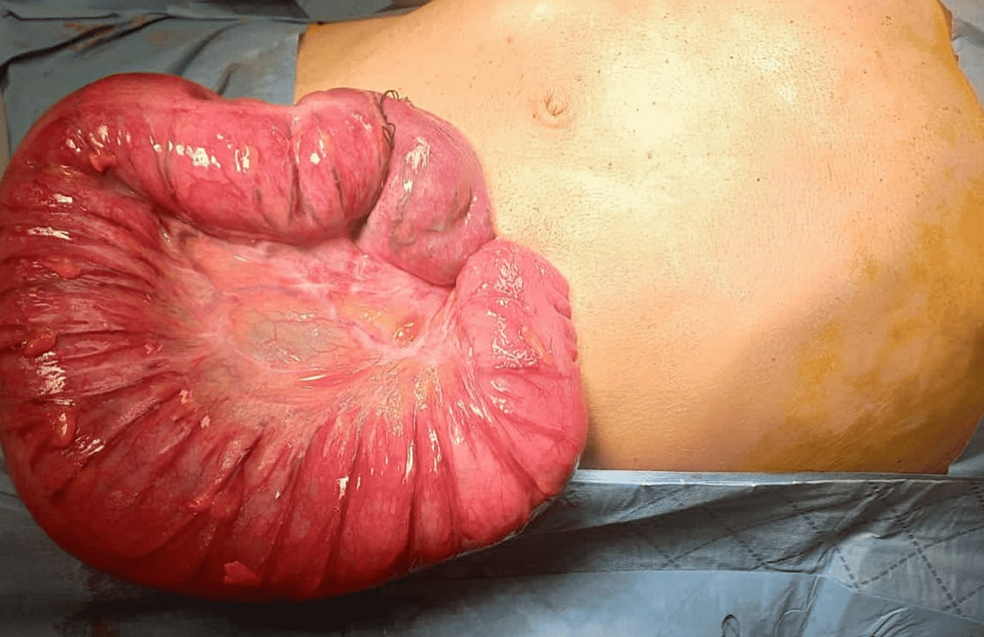
Sigmoid colon is exteriorized by gentle traction and viability is checked

Exteriorization by gentle traction of the dilated sigmoid colon was done and viability was checked to decide on either stoma formation or a primary anastomosis (Figure 1).

At this point, the Surgeon needs to make sure that all of the redundant colon is brought out to minimize the risk of recurrence. The vascular control can be achieved by serial ligation of vascular pedicles in a V shape or with the help of energy devices. The anastomosis is to be performed in either a manual fashion or with the help of a linear-stapling technique. Closure of the fascia is then performed with 2-0 braided absorbable sutures.

The last step is the purse-string circumferential subcutaneous suture, which is made by a 2-0 mono-filament non-absorbable suture, with a resulting defect of a maximum of 5mm diameter orifice. An iodine-soaked gauze was placed for 48 hours and then replaced by a dry gauze.

**Figure 2:**
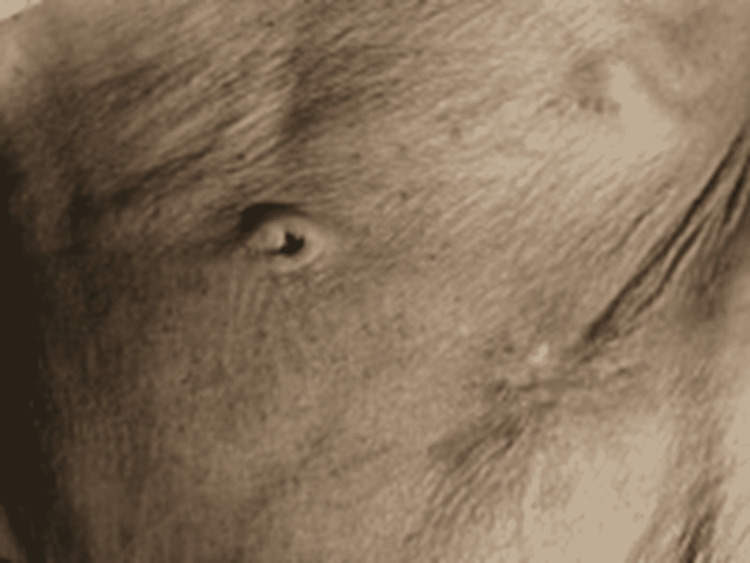
Post-operative aspect of the left iliac fossa mini-incision after six weeks

No surgical site infection was noted and the patient was pleased by the cosmetic result. The resulting scar in our case after six weeks is seen in Figure 2.

## Discussion

Sigmoid volvulus is a serious condition. In patients with clinical or radiological suspicion of gangrene or perforation, midline laparotomy is mandatory and should be performed as soon as the patient is fit for surgery. In patients with no complications, endoscopic detorsion is successful in up to 75% to 95 % of patients, with minimal morbidity. Definitive resection is mandatory to reduce the risk of recurrence [6-8].

As the sigmoid colon is redundant, exteriorization is generally easy through an off-midline incision. Left iliac fossa mini-incision has been proposed by some authors with variable success, with or without colonoscopic detorsion [3-5].

We present here a novel approach with a circular skin-disk incision, followed by a purse-string closure technique. We believe that first, it permits a more favorable cosmetic result, and second, as we know from the experience of stoma-reversal patients, it may result in lowering the incidence of surgical site infection [9].

## Conclusions

We highlight through this case report the feasibility of volvulus sigmoid resection through a skin-disk incision in the left iliac fossa without prior detorsion in fit patients without any clinical or radiological suspicion of gangrene or perforation. We propose the technique presented here as an alternative to the classical linear incision, as we think it may be associated with less surgical site infection incidence and a cosmetically more acceptable result.
